# Cell Death Pathways in Astrocytes with a Modified Model of Oxygen-Glucose Deprivation

**DOI:** 10.1371/journal.pone.0061345

**Published:** 2013-04-23

**Authors:** Qiaoying Huang, Rui Zhang, Liang yu Zou, Xu Cao, Xiaofan Chu

**Affiliations:** 1 Department of Neurology, People's Hospital of Shenzhen City, Second Clinical College, Jinan University, Shenzhen, China; 2 Research Centre for Neural Engineering, Institute of Biomedical and Health Engineering, Shenzhen Institutes of Advanced Technology, Chinese Academy of Sciences, Shenzhen, China; University of Kentucky, United States of America

## Abstract

Traditional oxygen-glucose deprivation (OGD) models do not produce sufficiently stable and continuous deprivation to induce cell death in the ischemic core. Therefore, we modified the OGD model to mimic the observed damage in the ischemic core following stroke and utilized this new model to study cell death pathways in astrocytes. The PO_2_ and pH levels in the astrocyte culture medium were compared between a physical OGD group, a chemical OGD group and a mixed OGD group. The mixed OGD group was able to maintain anaerobic conditions in astrocyte culture medium for 6 h, while the physical and the chemical groups failed to maintain such conditions. Astrocyte viability decreased and LDH release into in the medium increased as a function of exposure to OGD. Compared to the control group, the expression of active caspase-3 in the mixed OGD group increased within 2 h after OGD, but decreased after 2 h of OGD. Additionally, porimin mRNA levels did not significantly increase during the first 2 h of OGD, while bcl-2 mRNA levels decreased at 1 h. However, both porimin and bcl-2 mRNA levels increased after 2 h of OGD; interestingly, they both suddenly decreased at 4 h of OGD. Taken together, these results indicate that apoptosis and oncosis are the two cell death pathways responsible for astrocyte death in the ischemic core. However, the main death pathway varies depending on the OGD period.

## Introduction

Stroke is the second most common cause of death and is the leading cause of adult disability [1]–[3]. Once vessel occlusion occurs, the volume containing the impaired functionality is called the “core” region [4], [5]. As the brain cells within the core region die, primarily via necrosis, their intracellular contents diffuse into the surrounding extracellular space. This process leads to a secondary stage of cell damage, characterized by metabolic changes and eventual death, primarily via apoptosis, that progressively spreads to brain cells located in the ischemic penumbra [6], [7]. However, the core and penumbra continue to grow in volume as long as the ischemia is maintained. Notably, it is well known that subpopulations of astrocytes and neurons share a nearly identical set of channels and receptors. Morphologically, perivascular astrocytes are central to neurovascular units. Astrocytes directly communicate with neurons and dynamically interact with synapses through the uptake and release of neurotransmitters and receptor-mediated intracellular Ca^2+^ signaling [8]. Recent research has shown that oncosis may be the cell death pathway in astrocytes of the ischemic region, and oncotic astrocytes coexist with reactive astrocytes in the peripheral zone [9], [10]. However, the cell death pathway and the mechanism of these swollen astrocytes remain elusive.

Oxygen-glucose deprivation (OGD) is an in vitro model used to mimic the effects of stroke [11]. It is well suited to assessing the mechanism of action of drugs as well as the neurophysiological changes that occur with stroke. However, previous studies have exposed astrocytes to OGD in many different ways. Some used a culture medium containing Earle's solution without glucose and incubated in an anaerobic chamber filled with 95%N_2_ and 5%CO_2_ at 37°C [12], while others used an 85% N_2,_ 10% H_2_, and 5%CO_2_ atmosphere [13]. However, it has been difficult to rapidly and reliably lower the O_2_ concentration around cells to a desired level in these preparations. Another study used metabolic inhibitors (such as cyanide) to mimic O_2_ deprivation [14], but this method may not always be valid [15]. Some evidence indicates that hypoxia can be induced by directly and rapidly limiting O_2_ availability to all cellular activities using an oxygen scavenger (e.g., sodium hydrosulfite), but this technique does not maintain the long-term effects of O_2_ deprivation.

Therefore, it is of great interest to modify the OGD model to allow the in vitro assessment of the mechanism of astrocyte cell death in the ischemic core. Our study aims to improve the OGD model by providing rapid reduction in the O_2_ concentration around cells rapidly and maintaining the long-term effects of O_2_ deprivation to provide an in vitro model of the ischemic core. Furthermore, we demonstrate, for the first time, that astrocyte death in the ischemic core can proceed via separate yet complementary pathways, such as apoptosis and oncosis. In addition, oncosis may be the predominant cell death pathway involved in mediating astrocyte death following 3 h of anaerobic incubation.

## Materials and Methods

### Ethics statement

This study was carried out in strict accordance with the recommendations in the Guide for the Care and Use of Laboratory Animals of the National Institutes of Health. The protocol was approved by the Committee on the Ethics of Animal Experiments of Shenzhen Institutes of Advanced Technology (Permit Number: SIAT-IRB-110324- A0000). All surgery and all efforts were made to minimize suffering.

### Preparation and incubation of astrocytes

Astrocytes were obtained from neonatal Sprague-Dawley rats (less than 24 hours old) using a modified version of a previously described method [16]. Briefly, dissociated hippocampus were seeded into poly-l-lysine-coated 25 cm2 flasks (Corning, USA) and cultured in high glucose DMEM (Gibco, USA) supplemented with 10% fetal calf serum,10% neonatal calf serum (Gibco, USA) and 25 µg mL-1 penicillin- streptomycin (complete medium). The culture medium was transferred every 3 days. After approximately 7 days, the confluent cultures were shaken at 200 rpm and 37°C for 4 h to separate the astrocytes from the remaining microglia and oligodendroglia. The adherent cells were re-plated in complete medium and incubated in a humid atmosphere (5%CO_2_–95% air at 37°C) overnight. The next day, the adherent cells were trypsinized, resuspended in complete medium for 20 min and plated in the flasks. The purity of the cell culture was assessed by staining for glial fibrillary acidic protein (GFAP) (Sigma, USA). More than 95% of the cultured cells were GFAP-positive. The following experiments were performed using in vitro cultures between 18 and 21 days old, when they reached maximal sensitivity to OGD-induced cell death [17].

### Oxygen-glucose deprivation

First, the partial pressure of O2(PO2)and the pH of the serum- and glucose-free DMEM (Gibco, USA) were repeatedly measured using a blood-gas portable clinical analyzer (Abbott, i-STAT, USA) at different OGD time points (1 min, 30 min, 1 h) and under different concentrations of the oxygen scavenger sodium hydrosulfite (0.5 mM, 1 mM, 2 mM, 5 mM, 10 mM, 15 mM). During the measurements, the samples were incubated in a humid atmosphere (5% CO_2_–95% air at 37°C) and exposed to the atmosphere. Next, an optimal concentration of sodium hydrosulfite (10 mM) was found that clamped the PO_2_ reduction to zero and maintained the pH at a suitable vital cell range for nearly half an hour. Second, the astrocytes were divided into three groups: one with the addition of 10 mM sodium hydrosulfite exposed to the atmosphere (the chemical group), one with the addition of 10 mM sodium hydrosulfite incubated in an solution bubbled with 85% N_2_, 10% H_2_, and 5% CO_2_ gas (the mixed group) and one with no sodium hydrosulfite exposed to the same gas combination (the physical group). The PO_2_ and pH of the media among the three groups were repeatedly measure at different OGD time points (1 min, 30 min, 1 h, 2 h, 3 h, 4 h, and 6 h). Finally, the conditions that could best keep the PO_2_ at zero were deemed to be representative of the stroke core observed in vivo.

### Annexin V-FITC/PI double staining analysis by flow cytometry

Cellular double staining for annexin V and PI was determined using an Annexin V-FITC/PI Apoptosis Detection Kit (Merck, Germany). The astrocytes were exposed to mixed OGD for 0 h, 1 h, 2 h, 3 h, 4 h or 6 h. The kit detects vital, apoptotic and oncotic astrocytes induced by OGD. Approximately 1×10^6^ cells were then stained for 5 min at room temperature with annexin V and PI in a Ca^2+^ enriched binding buffer and analyzed by a Beckman Coulter flow cytometer. Annexin V-FITC and PI emissions were detected in the FL 1 and FL 2 channels of a FACScan flow cytometer using emission filters of 518 and 620 nm, respectively. Approximately 5000 counts were made for each sample. The proportions of vital (annexin V-FITC−/PI−), apoptotic (annexin V-FITC+/PI−), oncotic cells (PI+) were calculated using Cell Quest software [18], [19].

### Lactate dehydrogenase (LDH) leakage assay

As a marker of necrotic tissue damage, LDH release from damaged cells was determined by analyzing the incubation solution. LDH release was measured in the culture medium using a diagnostic kit (Jiancheng Bioengineering Institute, Nanjing, China). A small quantity of medium (40 µl) was transferred from the culture disks and analyzed by the kit. The total LDH activity was determined after freezing each culture at −80°C overnight and then rapidly thawing the cultures, thus inducing nearly complete cell damage. The percentage of LDH leakage was then calculated to determine membrane integrity.

### Histology

Paraformaldehyde (4%)-fixed astrocytes exposed to OGD for 0 h, 1 h, 2 h, 3 h, 4 h and 6 h were stained with the standard Hematoxylin–Eosin (Sigma, USA) according to the manufacturer's instructions. These samples were then observed under an optical microscope (Olympus CKX41-A32PH) (×200).

### Western blot analyses

Astrocyte samples were processed using Cell Lysis and Protein Extraction kits (Sigma, USA). A small amount of protein (30 µg) was separated with sodium dodecyl sulfate-polyacrylamide gel electrophoresis (SDS-PAGE), followed by a wet transfer onto a PVDF membrane (Beyotime, China). The membrane was incubated overnight at 4°C in Tris-Buffered Saline with 5% nonfat milk and 0.05% Tween-20 (TBST), followed by a 1 h incubation with an anti-active caspase-3 antibody (1∶1000; Abcam, UK) and an anti-porimin antibody (1∶100; Santacruz, USA) at room temperature (RT). The membrane was washed and incubated for 1 h at RT with horseradish peroxidase-conjugated anti-mouse (Jackson Immuno, USA) or anti-rabbit secondary antibodies (Jackson Immuno, USA) at a dilution of 1∶5000 in TBST. The membrane was then incubated in an enhanced chemiluminescence detection reagent (Pierce, UK).

### Electron microscopy

Electron microscopy was used to evaluate morphological changes as previously described [20]. After being detached from the culture dishes, cells were centrifuged at 800 rpm for 5 min, fixed with 2.5% glutaraldehyde in PBS at 4°C and washed three times with 6.8% Sabatini's solution (PBS with 6.8% sucrose). Samples were post-fixed with 2% buffered osmium tetroxide for 2 h at 4°C and washed three times in Sabatini's solution. The samples were then passed through a graded series of alcohol solutions (30, 50, 70, 90 and 100%) for 15 min each and through a graded series of acetone (90 and 100%) for 15 min each. This procedure was followed by treatment with propylene oxide (15 min), a 1∶1 Epon-acetone mix (2 h) and three changes in pure epon (twice for 3 h and overnight). Polymerization occurred overnight at 80°C. Ultrathin sections (50 nm) were cut with a Leicaultracut ultramicrotome, stained with lead citrate and uranyl citrate for 10 min each, and then examined and photographed with a transmission electron microscope (FEI Tecnai G2 12 Transmission Electron Microscope, The Netherlands). These procedures were undertaken at the electron microscopy facility at Ji Nan University.

### Preparation of total RNA and Quantitative RT-PCR

The cells were collected, and the total RNA was extracted using a PureLink RNA Mini Kit (Ambion, USA), according to the manufacturer's instructions. RNA concentration was estimated by absorbance at 260 nm using a UV spectrophotometer. The total RNA of each sample was reverse-transcribed into cDNA using a reverse transcription system (TaKaRa, Japan). The cDNA was amplified using the following primers: 5′-TTCAGCAACTACT-3′ (porimin-upstream), 5′-AACAC TATACCACCAACAA-3′ (porimin-downstream), 5′-TATGGAATCCTGTGGCATC-3′ (β-actin-upstream), 5′-GTGTTGGCATAGAGGTCTT-3′ (β-actin-downstream), 5′-TTTGTTACAGGGTTTCAT-3′ (Bax-upstream), 5′-ATATTATTGTCCAGTTCATC-3′(Bax-downstream), 5′-AGAACAGGGTATGATAAC-3′ (Bcl-2-upstream), or 5′-AGTCTTCATCTCCAGTAT-3′ (Bcl-2-downstream). These primers were designed using Beacon Designer 7 software. Amplifications were performed using a SYBR-green Premix Ex Taq kit (TaKaRa, Japan) under the following conditions: 50 cycles at 95°C for 30 s, 60°C for 30 s, and 72°C for 30 s. The PCR products were normalized relative to the levels of β-actin mRNA. Relative expression was determined using the following equation: fold induction2[ΔΔCt], where ΔΔCt = Ct gene of interest-Ct gene of β-actin.

### Fluorescence microscopy

Immunostaining was performed as previously described. Briefly, samples were fixed with 4% paraformaldehyde for 15 min at room temperature. The samples were pre-incubated with a blocking solution (10% neonatal goat serum or 10% fetal calf serum and 0.1% Triton X-100 in phosphate-buffered saline) for 30 min and then incubated with the following antibodies: a rabbit polyclonal anti-active caspase-3 antibody (1∶400, Sigma, USA) or anti-porimin antibody (1∶100, Santa Cruz, USA), or a mouse monoclonal anti-GFAP (1∶400, Sigma, USA) for 2 h at room temperature. Active caspase-3 or porimin and GFAP were visualized with the secondary antibody (Alexa 488 nm or 568 nm, Invitrogen, USA). The nucleus was visualized with diamidino-phenyl-indole (DAPI) (Beyotime, China). Images were acquired using a Leica confocal microscope.

### Statistical analysis

The data are presented as the mean ± SD from three independent experiments performed in quadruplicate or quintuplicate. Statistical analysis of the data was performed using Student's t test and ANOVA followed by Bonferroni's test. P values of 0.05 or less were considered to be statistically significant.

## Results

### Astrocyte cultures and the purity assay

Astrocytes were cultured from the hippocampus of 0- to 24-hour-old Sprague-Dawley rats, as previously described. The purity of the cell culture was verified when the proportion of cells co-stained with GFAP and DAPI was over 95% (shown in Fig. S1A&B).

### PO2 and pH levels in serum- and glucose-free DMEM with different concentrations of sodium hydrosulfite

**Figure 1:**
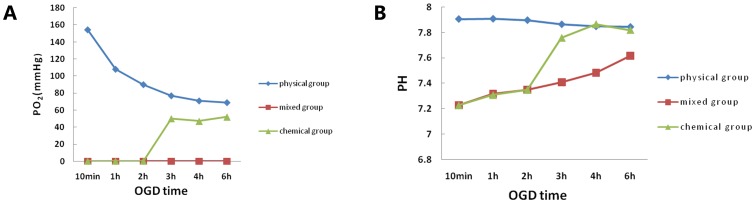
The PO_2_ and pH of the media among the three OGD groups. PO**_2_** (A) and pH (B) of the media among the three groups measured at different OGD time points. The mixed group kept the PO**_2_** at zero for 6 h in the incubation solution and mimics the ischemic core.

Our first objective was to find an optimal concentration of sodium hydrosulfite (Na_2_S_2_O_4_) to clamp PO_2_ reduction to zero and maintain the pH at a suitable vital cell range for nearly half an hour in the experimental atmosphere. The results of this calibration were presented in the Fig. S2, which showed that PO_2_ decreased gradually with the sodium hydrosulfite increasing. It had a dose-dependent relationship with sodium hydrosulfite, while 15 mM sodium hydrosulfite failed to maintain the pH of the OGD medium at an appropriate level. Then, 10 mM sodium hydrosulfite was the most appropriate concentration. Media with 10 mM sodium hydrosulfite can keep the PO_2_ at zero for 6 h in the incubation solution bubbled with 85% N_2_, 10% H_2_, and 5% CO_2_ gas, while the physical and the chemical groups failed to maintain such conditions (Fig. 1A&B).

### Astrocyte injury following exposure to mixed OGD

**Figure 2:**
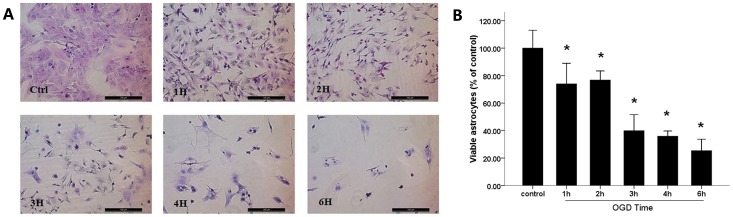
The viable astrocyte exposed to mixed OGD. (**A**) Astrocytes exposed to mixed OGD in the control condition (Ctrl) and for 1 h, 2 h, 3 h, 4 h and 6 h were stained by HE (200×). (B) Counting 5 views for statistical analysis, we found that the amount viable astrocytes decreased as a function of time spent under OGD. (*) indicates a significant difference (P<0.05) from the control group (Ctrl).

**Figure 3:**
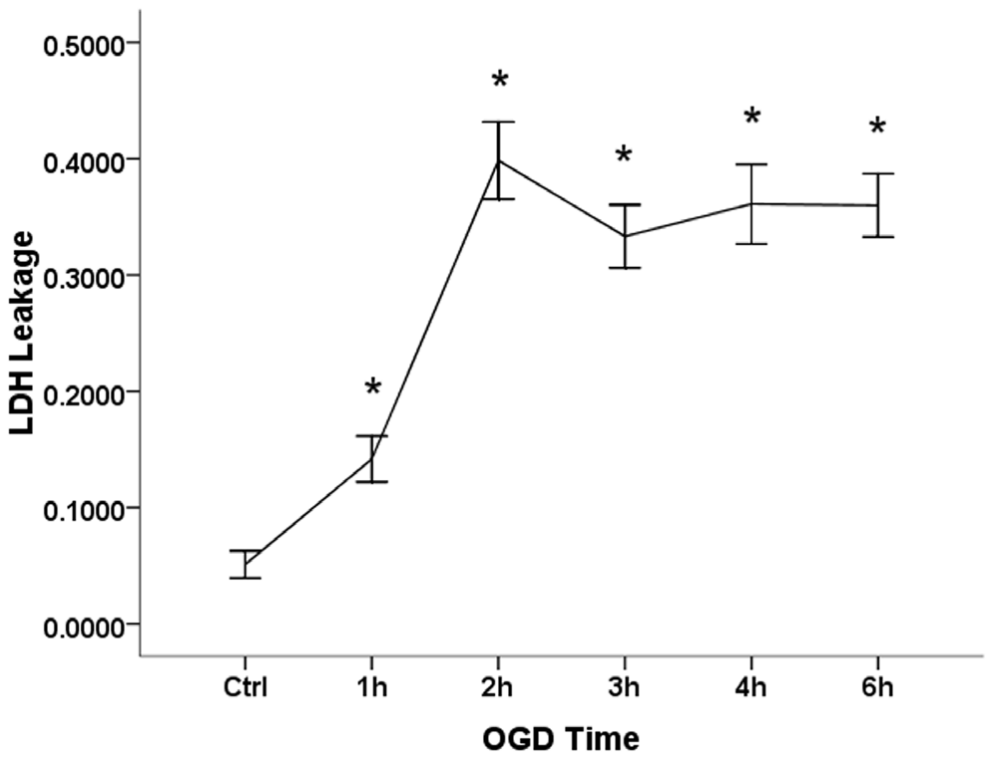
LDH leakage induced by mixed OGD. Mixed OGD induced a significant increase in LDH leakage after 1 h, 2 h, 3 h, 4 h and 6 h (F = 220.7, P = 0.001). (*) indicates a significant difference (P<0.05) from the control group (Ctrl).

**Figure 4:**
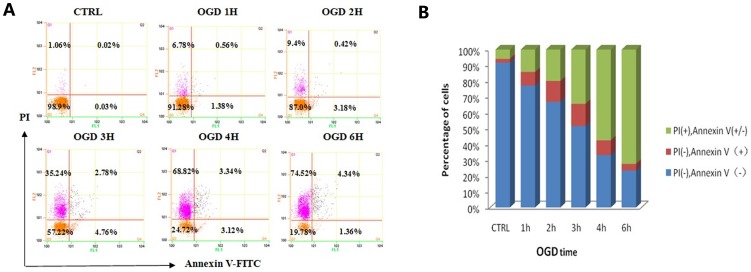
Detection of annexin V/PI-positive cells using FACS. (**A**) The effect of OGD on astrocyte death was quantified by detection of annexin V/PI-positive cells using FACS at different OGD time points. (B) Living (Annexin V−/PI−), apoptotic (Annexin V+/PI−) or oncotic (Annexin V+/PI+, Annexin V−/PI+) astrocytes were analyzed with 3 replications.

OGD led to severe astrocyte damage over the OGD time course, including swelling, vacuolization and hydropic degeneration accompanied by karyolysis, as observed under microscopy after HE staining, especially after 3 h of OGD. Counting 5 views for statistical analysis, we find that, compared to the control group, the viable astrocytes after OGD (1 h, 2 h, 3 h, 4 h and 6 h) significantly dropped to 74.00%±14.99%, 76.78%±6.63%, 39.91%±11.68%, 35.86%±3.84% and 25.38%±8.32% respectively. The number of viable astrocytes decreased as a function of time spent under OGD, especially after 3 h, the viable astrocytes reduced to less than half of the Control group. However, no significant difference was observed between 1 h and 2 h, or among 3 h, 4 h and 6 h (Fig. 2). Accordingly, OGD (1 h, 2 h, 3 h, 4 h and 6 h) induced a significant increase (16.68%±6.77%, 39.85%±6.07%, 33.07%±4.13%, 32.86%±7.62% and 35.98%±2.72% respectively) in LDH leakage (Fig. 3). However, the LDH leakage remained stable during the 3 h and 6 h time points. Propidium iodide and annexin V binding to externalized phosphatidylserine (PS) in the presence of a viability dye can be used to show the presence of apoptotic cells and oncotic cells derived from apoptosis. Cultures undergoing mixed OGD showed a decrease, compared to the control, in living astrocytes (annexin V−/PI−), a stable apoptotic population (annexin V+/PI−) and a rapid increase in the oncotic cells (annexin V+/PI+, annexin V−/PI+) assayed by the annexin V and PI kit (Fig. 4).

### Western blots for active caspase-3

**Figure 5:**
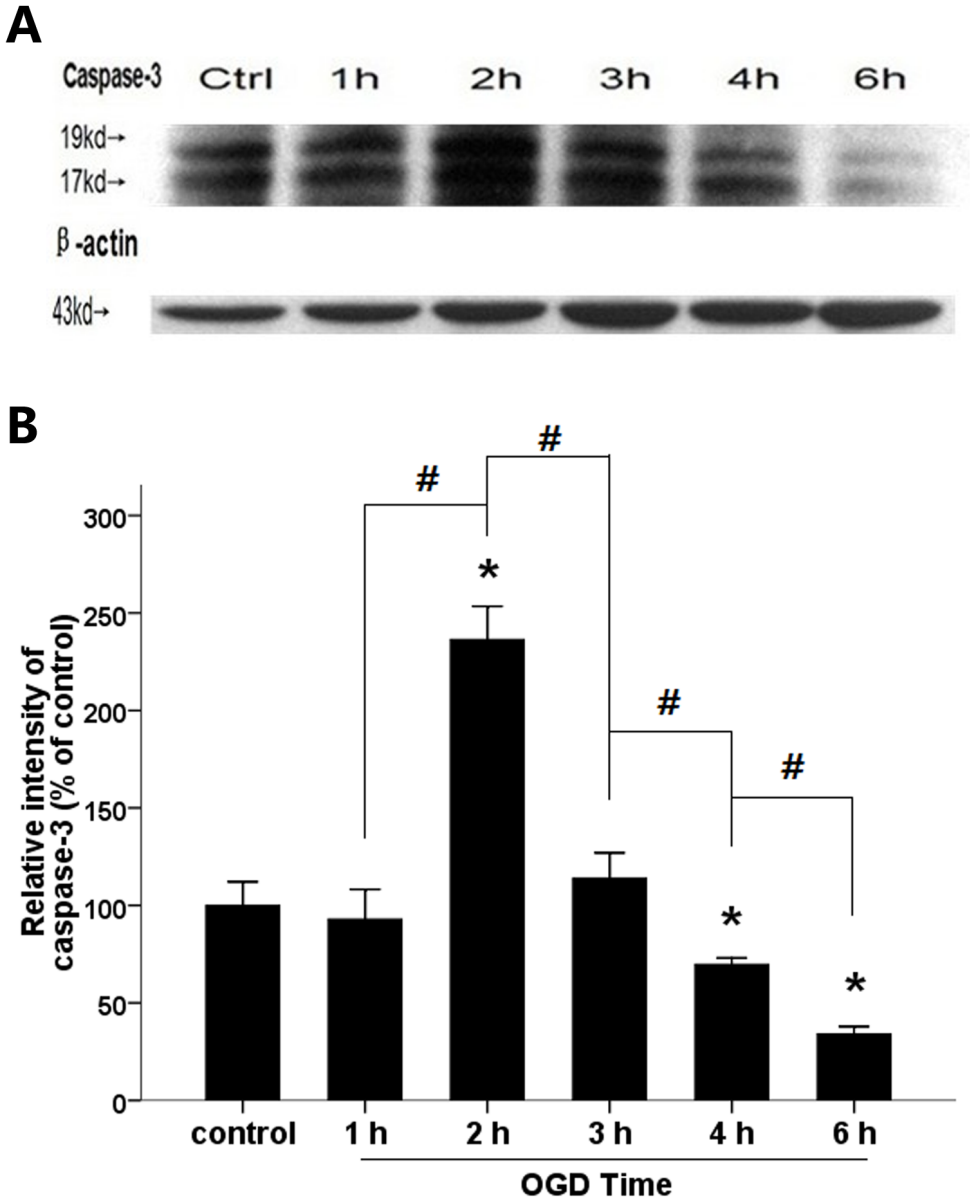
Western blot about active caspase-3. (A) Representative Western blot for active caspase-3 in astrocytes exposed to mixed OGD for 0 h (Ctrl), 1 h, 2 h, 3 h, 4 h and 6 h. (B) Relative density of the proteins measured by quantity one. Data are expressed as the mean± SD; (*) indicates a significant difference (P<0.05) from the control group; (#) indicates a significant difference between two adjacent OGD time points.

Increasing the mixed OGD time from 0 h to 2 h increased active caspase-3 protein levels, while increasing the mixed OGD time from 4 h to 6 h decreased active caspase-3 protein levels (69.75%±3.27%, 34.05%±3.81% versus control, P<0.05) (Fig. 5).

### Quantitative RT-PCR of bcl-2 and porimin mRNAs

**Figure 6:**
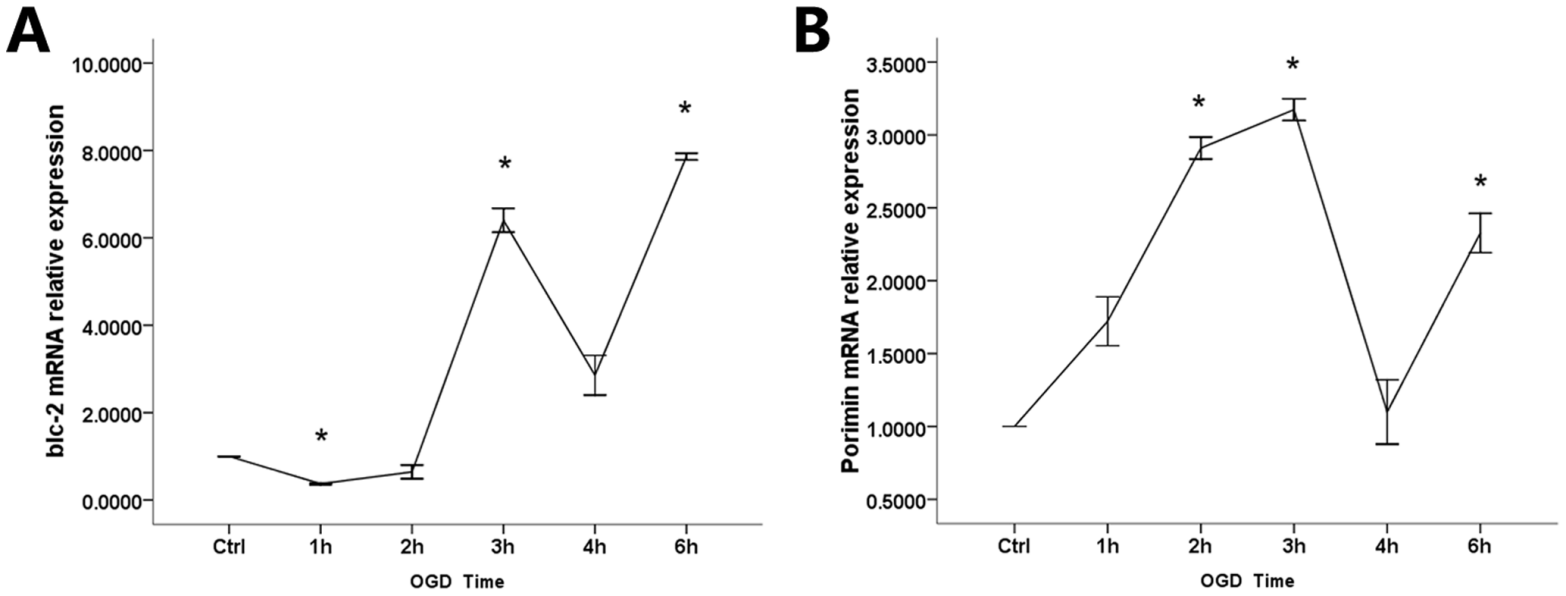
mRNA levels of bcl-2 and porimin in astrocytes exposed to mixed OGD. (A) the bcl-2 mRNA relative expression and (B) porimin mRNA relative expression for 1 h, 2 h, 3 h, 4 h and 6 h were altered relative to the rates of expression in the control samples (0 h). (*) indicates a significant difference (P<0.05) from the control group.

Analysis of porimin mRNA levels in astrocytes exposed to mixed OGD reveals a time-dependent increase, starting at 2 h (291.00%±7.59% versus control, P<0.05), peaking at 3 h (317.34%±7.42% versus control, P<0.05) and declining at 4 h (109.91%±22.08% versus control, P>0.05); the bcl-2 mRNA levels also reached a peak at 3 h and 6 h (640.23%±27.27%, 785.85%±7.54% versus control, P<0.05), but were lower than the control at 1 h (37.16%±2.00% versus control, P<0.05) (Fig. 6).

### Ultrastructural changes in astrocytes observed by fluorescence microscopy

Dying cells were distinguished by morphologic and biochemical traits and can be categorized into two distinct pathways: apoptosis and oncosis. Apoptosis was an energy-dependent process that was morphologically characterized by cell shrinkage, whereas oncosis was defined as a pre-lethal pathway leading to cell death that was associated with cellular swelling, organelle swelling and increased membrane permeability [21], [22]. In agreement with our previous reports [10], apoptosis and oncosis were involved in astrocyte death at the same time in the ischemic core. However, the main death pathways were not at equilibrium during different OGD time points. Oncosis may be the main death pathway of astrocytes in the ischemic core region during mixed OGD at later time points.

## Discussion

According to our previous study, neither the chemical OGD model nor the physical OGD model could produce sufficiently stable and continuous oxygen deprivation to mimic cell death in the ischemic core (shown in Fig. 1A and Fig. S3&S4) .Therefore, we designed a method that precisely clamps the O_2_ level around cells at zero and maintains a relatively constant PO_2_ for an extended period of time using sodium hydrosulfite. Sodium hydrosulfite induced hypoxia by directly limiting the O_2_ available for cellular activities according to the following chemical reactions [23]: 2Na_2_S_2_O_4_+O_2_+2H_2_O→4·NaHSO_3_; 2·NaHSO_3_ + O_2_→4·NaHSO_4_.

There are two important features of our method. (1) With the addition of sodium hydrosulfite (10 mM), the PO_2_ around the cells quickly reaches zero and stays at that level for an extended period of time (at least 6 h) (Fig. 1A), which mimics the ischemic core. Another study also found that sodium hydrosulfite (1 mM) had a similar acute effect, but the subsequent PO_2_ levels quickly increased [24]. (2) PO_2_ has a dose-dependent relationship with sodium hydrosulfite (Fig. S2). The PO_2_ decreased gradually with the sodium hydrosulfite increasing. As mentioned above, the use of sodium hydrosulfite greatly enhances our ability to control PO_2_, which may be used to mimic the ischemic core or even the ischemic penumbra. This method enables us to study the response of co-cultured or isolated cells to discrete changes in O_2_ availability. Furthermore, an additional advantage of this technique is that it is a fast, reliable method to induce oxygen-glucose deprivation and reperfusion.

Because oxygen deprivation was induced using a chemical oxygen scavenger, the possible side effects of sodium hydrosulfite need to be considered. One of our concerns with using sodium hydrosulfite is that the byproducts of the reactions, sodium bisulfite and sodium bisulfate, are acidic. Therefore, we also examined the effect on pH in this study. We found that the addition of 10 mM sodium hydrosulfite to glucose-free DMEM, bubbling with room air, results in the pH coming to a final value of 7.45 after 1 h. Notably, the pH of the OGD medium can maintain a level between 7.2 and 7.6 in the presence of 10 mM sodium hydrosulfite throughout the OGD time course (Fig. 1B), while 15 mM sodium hydrosulfite failed. As the acid shift depended on the amount of CO_2_ consumed, the OGD medium was equilibrated by incubating with 85% N_2_, 10% H_2_ and 5% CO_2_ gas [25]. To determine whether sodium hydrosulfite induced other changes in cell properties, we also examined the osmotic pressure of the OGD medium containing 10 mM sodium hydrosulfite and found that the osmotic pressure was still in the normal range (data not shown). Therefore, the mixed OGD model is a stable oxygen-glucose deprivation model that can imitate injury within the ischemic core. A significant advantage of this model is that rapid, reversible and reproducible changes can be made in the O_2_ level around cells.

Furthermore, we verified that the mixed OGD model can cause acute cell injury using primary culture astrocytes. The astrocytes acquired further injuries as a function of time spent in the mixed OGD condition. Compared with the control group, the cell survival rate decreased as OGD-treatment continues. The amount of adherent astrocytes stained by HE decreased as a function of time spent under OGD especially after 3 h (Fig. 2). After incubation in OGD for 3 h, nearly 50% of the astrocytes were dead, indicated by PI (+) (Fig. 4). Similar results were shown for LDH release. We observed that LDH release peaks 2 h after exposure to OGD, when almost 40% of the LDH had leaked out through the injured membrane, and remained high throughout the experimental period. The peak indicates that astrocyte death proceeds via more than one pathway throughout the time course of OGD treatment and that oncosis may play an important role. These data indicate that OGD insult could elevate cell death and reduce cell viability, which mimics the ischemic core in vivo.

**Figure 7:**
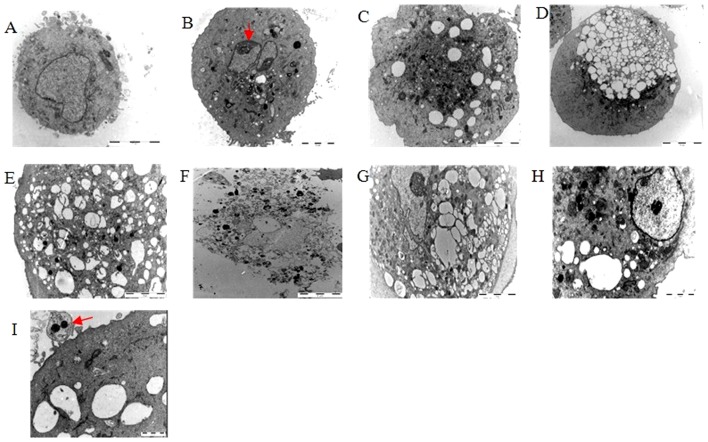
Representative electron microscopy images of the ultrastructural changes in astrocytes induced by mixed OGD. (A) Image of a normal astrocyte (6200×). (B,I) Images of typical apoptotic cells (the red arrowhead shows chromatin condensation rendering a curved profile to the nucleus and apoptotic body formation). Apoptotic cells were found in almost all samples, particularly after 1 h (B) and 2 h (C) of OGD. (G–H) Images of typical oncotic-like cells. The co-existence of cellular swelling and vacuolization were found in the 3 h (D) and 4 h (E) OGD samples. After 6 h of OGD, the astrocytes were almost fragmental (F). Scale bars: I: 1 µm; H: 2 µm; D: 10 µm; all others: 5 µm.

Using electron microscopy images acquired from astrocytes exposed to the mixed OGD condition (Fig. 7), we observed increasing astrocyte death as a function of time spent under OGD, particularly after 3 h and 4 h, characterized by mitochondrial swelling, dilation of the endoplasmic reticulum and Golgi, increased membrane permeability, cellular swelling and vacuolization, and advanced cytoplasmic degradation and karyolysis. However, we also found that some of the astrocytes died due to apoptosis, as shown by cell shrinkage and chromatin condensation, which rendered a curved profile to the nucleus and apoptotic body formation (Fig. 7B and 7I). We also found that nearly all cells were fragmented after 6 h of OGD. Both apoptosis and oncosis can lead to necrosis through postmortem autolytic and degradative changes [22], and each of these morphological changes appeared in cells during different periods of OGD in the present study, which indicates that the astrocyte death pathway in the ischemic core may not be limited to apoptosis but may also include another type of cell death similar to oncosis. We next sought to determine which of the two pathways are predominantly involved in mediating mixed OGD-induced astrocyte death and how each pathway functionally contributes to cell death.

As shown in Fig. 4, the viability of the astrocytes rapidly decreased and the number of astrocytes stained by propidium iodide increased as a function of time spent under OGD. Approximately 50% of the astrocytes remained viable and more than 30% took up propidium iodide when incubated for 3 h under anaerobic conditions. The percentage of astrocytes that lacked membrane integrity was assessed by the number of cells positively stained for propidium iodide. The apoptotic astrocytes, marked as annexin V positive and propidium iodide negative, remained stable during the OGD period. The proportion of astrocytes in which membrane permeability increased was greater than that of the apoptotic astrocyte population at 3 h. As for protein expression, the levels of active caspase-3 in the mixed OGD group increased within 2 h after OGD, but decreased after 3 h of OGD (Fig. 5).It indicates that astrocyte death proceeds via more than one pathway (such as apoptosis) throughout the time course of OGD treatment and oncosis may play an important role at late stage following OGD.

Active caspase-3 is considered to be a specific marker of apoptosis [26], and porimin may be specific for oncosis [27]. As shown in Fig. 6, the OGD treatment produced oncosis that up-regulated the expression of the apoptosis inhibitor gene bcl-2, which can inhibit permeability transition (PT), the release of apoptogenic proteins from mitochondria [28], and the expression of the pro-oncotic gene porimin at late time points following OGD. With persisting OGD, cellular energy depletion following metabolic insults results in a reduction of mitochondrial respiration and ATP synthesis [21]. Evidence from our previous study suggests that intracellular ATP levels can determine the cell-death fate by apoptosis or oncosis [29] and that depletion of intracellular ATP can irreversibly induce oncotic cell death. Once the cellular ATP content is depleted to less than 35% of the control, astrocytes die primarily through oncosis [26]. Thus, oncosis is the predominant pathway of astrocytic cell death during late OGD because of the severe ATP depletion. Taken together, these results indicate that the astrocytes in the ischemic core undergo not only apoptotic but also oncotic and other cell death pathways during acute OGD. Interestingly, the expression of bcl-2 and porimin mRNAs both suddenly decreased after 4 h of OGD in our present study, suggesting an unknown feedback mechanism that requires future investigation.

In conclusion, we demonstrate for the first time that a mixed OGD model can induce stable and continuous deprivation for 6 h, a method which mimics the ischemic core in vivo. In this study, astrocyte death induced by the mixed OGD model was a consequence of separate yet complementary pathways, i.e., apoptosis and oncosis. In addition, the predominant cell death pathway varied over the course of OGD treatment.

## Supporting Information

Figure S1 **Astrocyte cultures.** (A) The cultured astrocytes were assessed with GFAP (green) and DAPI (blue); over 95% of the cells were astrocytes (400×). (B) The morphology of the astrocytes was observed using a Leica confocal microscope (1200×).
(TIF)

Figure S2 **The PO_2_ and pH of different concentrations of sodium hydrosulfite.** PO**_2_** (A) and pH (B) of the serum- and glucose-free DMEM (Gibco, USA) with the addition of different concentrations of sodium hydrosulfite to the atmosphere.
(TIF)

Figure S3 **The viable astrocyte between the mixed OGD and the physical OGD model.** (A) Astrocytes exposed to mixed and the physical OGD model in the control condition (Ctrl) and for 1 h, 2 h, 3 h, 4 h and 6 h were stained by HE (200×). (B) Counting 5 views for statistical analysis, we found that the amount viable astrocytes were different between the two models as a function of time spent under OGD. Data are expressed as the mean ± SD; (*) indicates a significant difference (P<0.05) between the mixed group and the physical group.
(TIF)

Figure S4 **The LDH leakage between the mixed OGD and the physical OGD model.** The LDH leakage of astrocytes exposed to mixed and the physical OGD model in the control condition (Ctrl) and for 1 h, 2 h, 3 h, 4 h and 6 h. Data are expressed as the mean ± SD; (*) indicates a significant difference (P<0.05) between the mixed group and the physical group.
(TIF)
