# Supplementary figures and images for: Cell Death Pathways in Astrocytes with a Modified Model of Oxygen-Glucose Deprivation
Source: PLoS One. 2013 Apr 23;8(4):e61345. doi: 10.1371/journal.pone.0061345 (PMC3634069; doi:10.1371/journal.pone.0061345)

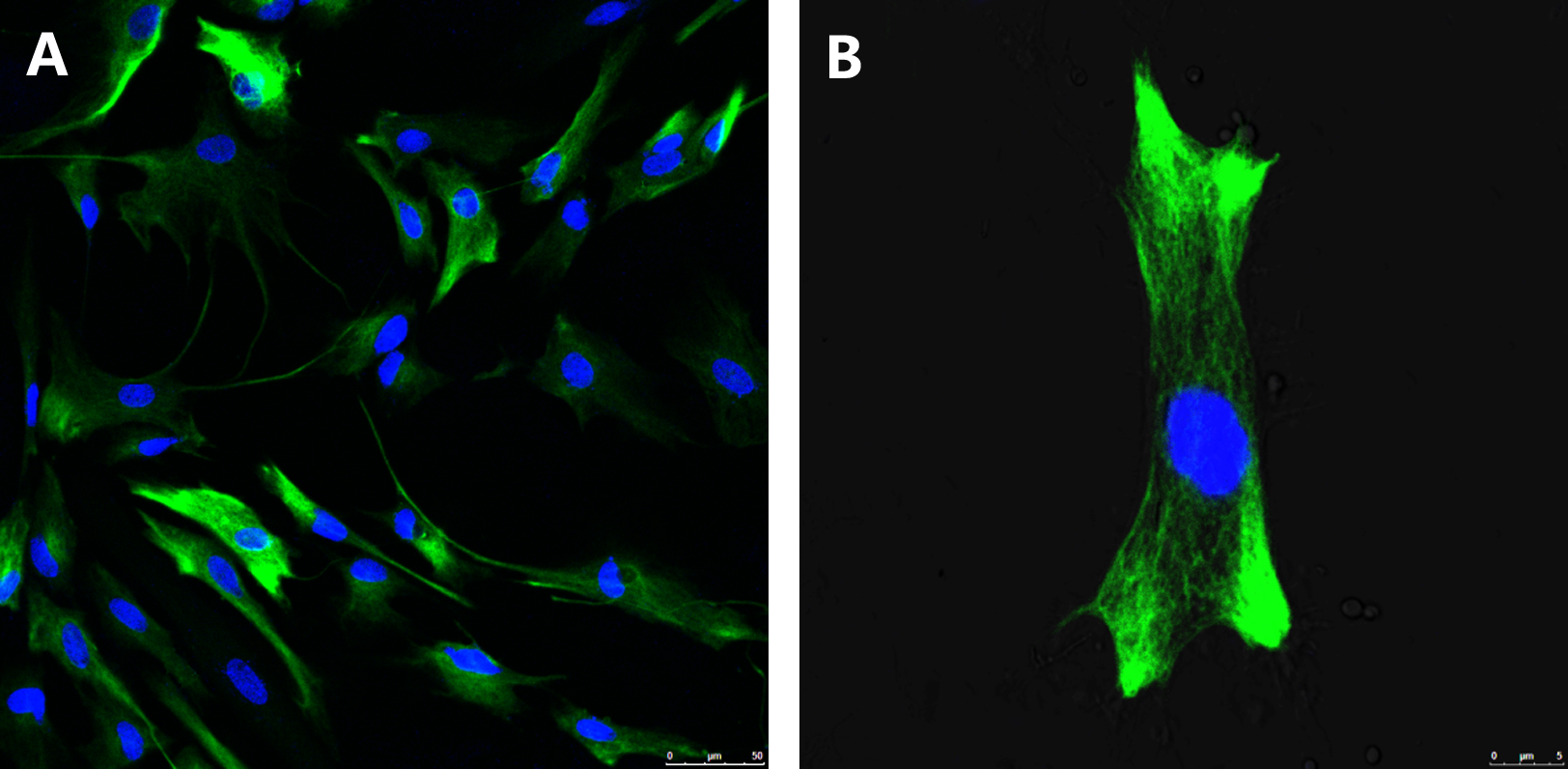

Supplement: Figure S1 — Astrocyte cultures. (A) The cultured astrocytes were assessed with GFAP (green) and DAPI (blue); over 95% of the cells were astrocytes (400×). (B) The morphology of the astrocytes was observed using a Leica confocal microscope (1200×). (TIF) [file pone.0061345.s001.tif]

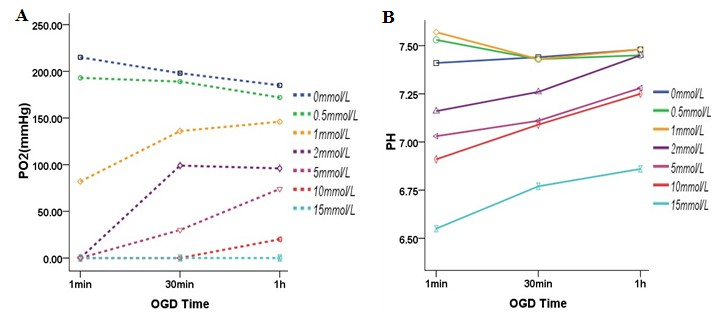

Supplement: Figure S2 — The PO2 and pH of different concentrations of sodium hydrosulfite. PO2 (A) and pH (B) of the serum- and glucose-free DMEM (Gibco, USA) with the addition of different concentrations of sodium hydrosulfite to the atmosphere. (TIF) [file pone.0061345.s002.tif]

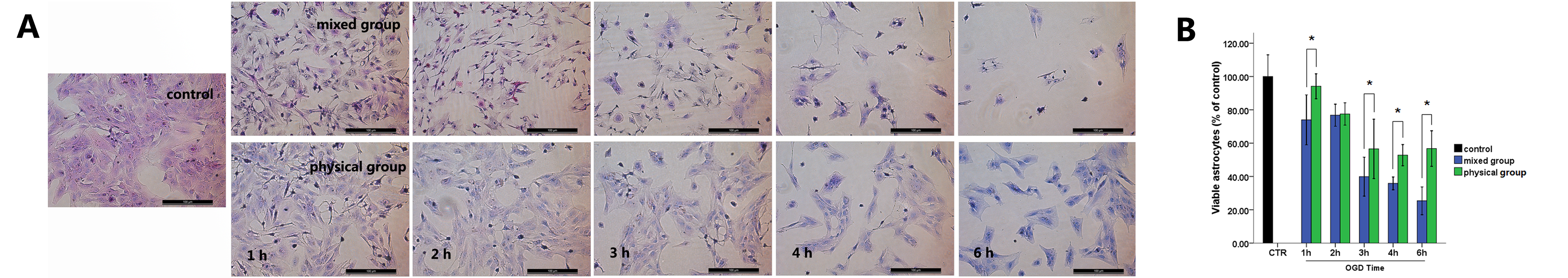

Supplement: Figure S3 — The viable astrocyte between the mixed OGD and the physical OGD model. (A) Astrocytes exposed to mixed and the physical OGD model in the control condition (Ctrl) and for 1 h, 2 h, 3 h, 4 h and 6 h were stained by HE (200×). (B) Counting 5 views for statistical analysis, we found that the amount viable astrocytes were different between the two models as a function of time spent under OGD. Data are expressed as the mean ± SD; (*) indicates a significant difference (P<0.05) between the mixed group and the physical group. (TIF) [file pone.0061345.s003.tif]

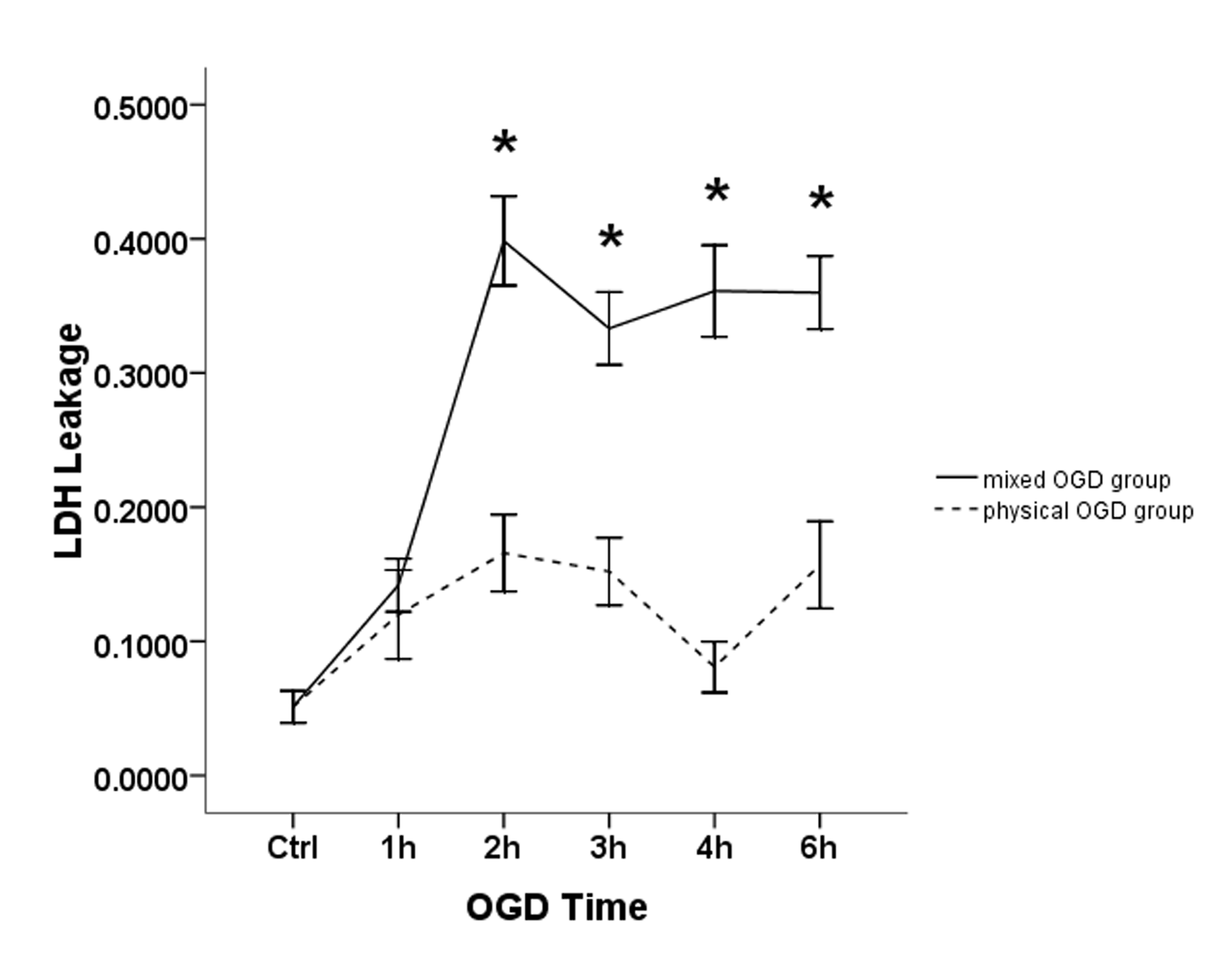

Supplement: Figure S4 — The LDH leakage between the mixed OGD and the physical OGD model. The LDH leakage of astrocytes exposed to mixed and the physical OGD model in the control condition (Ctrl) and for 1 h, 2 h, 3 h, 4 h and 6 h. Data are expressed as the mean ± SD; (*) indicates a significant difference (P<0.05) between the mixed group and the physical group. (TIF) [file pone.0061345.s004.tif]
